# StainNet: A Fast and Robust Stain Normalization Network

**DOI:** 10.3389/fmed.2021.746307

**Published:** 2021-11-05

**Authors:** Hongtao Kang, Die Luo, Weihua Feng, Shaoqun Zeng, Tingwei Quan, Junbo Hu, Xiuli Liu

**Affiliations:** ^1^Britton Chance Center for Biomedical Photonics, Wuhan National Laboratory for Optoelectronics, Huazhong University of Science and Technology, Wuhan, China; ^2^Ministry of Education (MOE) Key Laboratory for Biomedical Photonics, School of Engineering Sciences, Huazhong University of Science and Technology, Wuhan, China; ^3^Department of Pathology, Hubei Maternal and Child Health Hospital, Wuhan, China

**Keywords:** stain normalization, cytopathology, histopathology, convolutional neural network (CNN), generative adversarial network (GANs)

## Abstract

Stain normalization often refers to transferring the color distribution to the target image and has been widely used in biomedical image analysis. The conventional stain normalization usually achieves through a pixel-by-pixel color mapping model, which depends on one reference image, and it is hard to achieve accurately the style transformation between image datasets. In principle, this difficulty can be well-solved by deep learning-based methods, whereas, its complicated structure results in low computational efficiency and artifacts in the style transformation, which has restricted the practical application. Here, we use distillation learning to reduce the complexity of deep learning methods and a fast and robust network called StainNet to learn the color mapping between the source image and the target image. StainNet can learn the color mapping relationship from a whole dataset and adjust the color value in a pixel-to-pixel manner. The pixel-to-pixel manner restricts the network size and avoids artifacts in the style transformation. The results on the cytopathology and histopathology datasets show that StainNet can achieve comparable performance to the deep learning-based methods. Computation results demonstrate StainNet is more than 40 times faster than StainGAN and can normalize a 100,000 × 100,000 whole slide image in 40 s.

## Introduction

Tissues or cells are usually transparent and need to be stained before observation under a microscope. However, the potential factor in the staining reagent, staining process, and slide scanner specifications often result in inconsistency of pathological images (1). These variations not only affect the judgment of pathologists but also weaken the performance of CAD systems and hamper their applications in pathology (2–4). So, stain normalization is a routine pre-processing operation for pathological images, especially for CAD systems, and it is reported to help increase the prediction accuracy, such as tumor classification (5). Stain normalization algorithms usually transfer the color style of the source image to that of a target image (6) while preserving the other information in the processed image (7), which can be broadly classified into two classes: conventional methods and deep learning-based methods.

Conventional methods are mainly realized by analyzing, converting, and matching color components, which can be divided into color matching and stain-separation methods. Color matching methods calculated the mean and SD of source images and matched them to a reference image in the Lab color space (8, 9). Stain-separation methods try to separate and normalize each staining channel independently (10–12). For instance, Ruifrok and Johnston (10) proposed to measure the relative proportion for three channels (R, G, and B) with the slides stained by only a single stain reagent (Hematoxylin or Eosin) to estimate stain vectors. And different mathematical methods were applied to compute stain vectors, such as singular value decomposition (SVD) in Optical Density (OD) space (11), sparse non-negative matrix factorization (SNMF) (12), or a pertained classifier (6). However, Pap stain used in cervical cytopathology involves not only Hematoxylin and Eosin but also Orange, Light Green, and Bismarck Brown (13), which makes it more difficult to distill the various dye vectors on cervical cytopathology. Nevertheless, most of these methods rely on a reference image to estimate stain parameters, but it is hard for one reference image to cover all staining phenomena or represent all input images, which usually causes misestimation of stain parameters and thus delivers inaccurate normalization results (14, 15).

Deep learning-based methods mostly apply generative adversarial networks (GANs) to achieve stain normalization (3, 7, 8, 16–18). Shaban et al. (8) proposed an unsupervised stain normalization method named StainGAN based on CycleGAN (16) to transfer the stain style. Cai et al. (3) proposed a new generator to improve the image quality and accelerate the networks. On the other hand, Cho et al. (18), Salehi et al. (7), and Tellez et al. (17) reconstructed original images from the images with color augmentations, e.g., grayscale and Hue-Saturation-Value (HSV) transformation, and tried to normalize other color styles to the original. However, due to the complexity of deep neural networks and the instability of GANs, it is hard to preserve all source information; sometimes, it has a risk of introducing some artifacts, which has some adverse effects on subsequent analysis (19). At the same time, the network of deep learning-based methods usually contains millions of parameters, so it generally requires high-computing resources and the computing efficiency is generally low (14).

Deep learning-based methods perform well in stain normalization, but they are not satisfactory in the robustness and computational efficiency. In this paper, we propose a stain normalization network named StainNet, which employs a fully 1 × 1 convolution network to adjust the color value in a pixel-by-pixel manner. In the method, StainGAN was used as the teacher network and StainNet as the student network to learn the color mapping by distillation learning. Results show that StainNet can achieve comparable normalization performance with StainGAN but retains the source information better. The results also demonstrate that StainNet was more than 40 times faster than StainGAN in computational efficiency, which allows StainNet to normalize a 100,000 × 100,000 whole slide image in 40 s.

## Materials and Methods

### Dataset

Five datasets were used to evaluate the performance of different methods. Among them, the aligned cytopathology dataset and the aligned histopathology dataset are used to evaluate the similarity between the normalized image and the target image. The cytopathology classification dataset and the histopathology classification dataset are used to verify normalization algorithms in the classification task. Twenty metastases whole slide images (WSIs) from the University Medical Center Utrecht in Camelyon16 testing part was used to test the effects of the StainNet normalization on the clinical diagnostics. This study was approved by the Ethics Committee of Tongji Medical College, Huazhong University of Science and Technology.

#### The Aligned Cytopathology Dataset for Evaluating the Similarity

These cytopathology datasets are taken from the same slides (Thinprep cytologic test slides from the Maternal and Child Hospital of Hubei Province) with two slide scanners. One scanner is custom constructed, called Scanner O, equipped with a 20x objective lens with a pixel size of 0.2930 μm. The other from Shenzhen Shengqiang Technology Co., Ltd., called scanner T, has a 40x objective lens and a pixel size of 0.1803 μm. We resampled the images from scanner T to reduce the pixel size to 0.2930 μm, and then performed rigid and no-rigid registration to align the resampled images to these from scanner O. Finally, 3,223 aligned image pairs with the size of 512 × 512 pixels were collected. Among these images, 2,257 pairs of images were randomly selected as the training set, and the remaining 966 pairs of images were used as the test set. The images from the scanner O and T are seen as source images and target images, respectively.

#### The Cytopathology Classification Dataset for Verifying Normalization Algorithms

This dataset used the same data source as that in section The Aligned Cytopathology Dataset for Evaluating the Similarity. The patches from scanner T are used as the training set to train the classifier, and these from scanner O are used as the test set to evaluate the classifier. In this dataset, the patches with abnormal cells were labeled by cytopathologists as abnormal patches and the patches without abnormal cells as normal patches. There are 6,589 abnormal patches, 6,589 normal patches in the training dataset, 3,343 abnormal patches, and 3,192 normal patches in the test dataset. The resolution of patches was resampled to 256 × 256 with 0.4862 μm per pixel. We used StainGAN and StainNet trained on the aligned cytopathology dataset in section The Aligned Cytopathology Dataset for Evaluating the Similarity to normalize the patches in the test set to the style of the training set. Then, we used the original test set and the normalized test set to verify the necessity of stain normalization and evaluate the performance of StainGAN and StainNet.

#### The Aligned Histopathology Dataset for Evaluating the Similarity

The histopathology dataset is from the publicly available part of the MITOS-ATYPIA ICPR'14 challenge (20). In the MITOS-ATYPIA dataset (20), there are 16 slides with standard hematoxylin and eosin (H&E) staining, 11 slides as the training set, and 5 slides as the test set. And all the aligned images are taken from the same slide but using two slide scanners: Aperio Scanscope XT called scanner A and Hamamatsu Nanozoomer 2.0-HT called scanner H. The number of image frames is variable from slide to slide. The training data set contains 1,200 frames, and the test data set contains 496 frames at 40x magnification. The resolution of the frames from scanner H was resampled to that of frames from scanner A, and then performed rigid and no-rigid registration to align the resampled frames to these from scanner A. We cropped 16 patches with the size of 256 × 256 from every frame without overlap, so there are 19,200 patch pairs in our training set and 7,936 patch pairs in our testing set. In this dataset, the images from the scanners A and H are seen as source images and target images, respectively.

#### The Histopathology Classification Dataset for Verifying Normalization Algorithms

The publicly available Camelyon16 dataset (21) is used, which contains 399 WSIs from two centers. In our experiments, 170 WSIs from Radboud University Medical Center in Camelyon16 training part were used to extract the training patches, and 50 WSIs from University Medical Center Utrecht in Camelyon16 testing part were used to extract the test patches. We labeled the patches containing tumor cells as abnormal and the patches not containing any tumor cells as normal. For abnormal patches, we extracted patches of size 256 × 256 from the tumor area in tumor slides. For normal patches, we randomly extracted patches of size 256 × 256 from the normal area in tumor slides and normal slides until the number of normal patches was equal to the number of abnormal patches. In this way, there are 40,000 patches in our training set and 10,000 patches in the testing set. In addition, we also randomly extracted 6,000 patches from the training set and test set to train StainGAN and StainNet, where the patches from the test set were used as the source image, and the patches from the training set were used as the target image. For the classifier trained on the training set, we used the original test set and the normalized test set to evaluate the classifier and the performance of StainGAN and StainNet.

### StainNet for Stain Normalization

**Figure 1:**
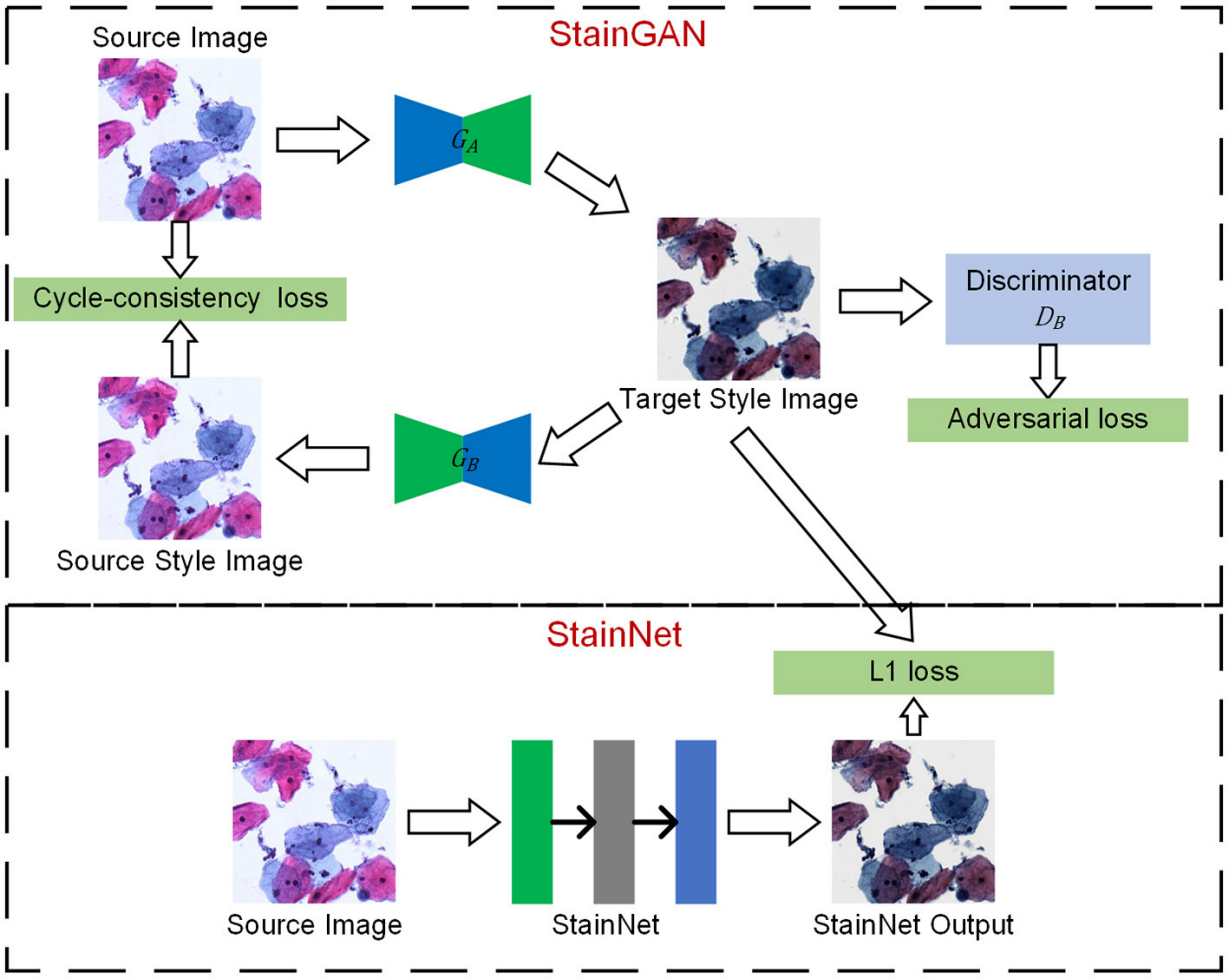
The framework of StainNet. First, StainGAN normalizes the images from the source domain to the target domain. Then, the normalized images by StainGAN are set as Ground Truth to train StainNet. The images from the source domain are mapped to the source domain and then back to the target domain by StainGAN. The same reverse process is also performed for images from the target domain. StainNet is a fully 1 × 1 convolutional neural network, which can directly map the images from the source domain to the target domain.

The framework is shown in Figure 1, which mainly consists of two steps: one step is StainGAN training, a generative confrontation network with two generators and two discriminators, and the other step is StainNet generation, which is composed of a fully convolutional neural network. StainNet needs paired source and target images to learn the transformation from the source color space to the target color space. In practice, it is hard to get the paired images and align the images perfectly; we used StainGAN as the teacher network and StainNet as the student network. That is, StainNet uses the L1 loss to learn the output of StainGAN.

There are two generators (*G*_*A*_ and *G*_*B*_) and a discriminator (*D*_*A*_ and *D*_*B*_) in StainGAN. *G*_*A*_is used to transfer the image from the source domain to the target domain, and *G*_*B*_ is used to transfer from the target domain to the source domain. *D*_*A*_ is used to distinguish the image generated by *G*_*A*_and a real target image, *D*_*B*_is used to distinguish the image generated by *G*_*B*_ or a real source image. There are two losses in StainGAN, namely cycle-consistency loss and adversarial loss. The cycle-consistency loss (16) ensures that the generated images by *G*_*A*_ can be reconstructed to source image by *G*_*B*_, and the generated images by *G*_*B*_ can be reconstructed to target image by *G*_*A*_. The adversarial loss tries to ensure the stain distribution of the generated images is consistent with the real distribution.

In the current convolutional neural network, convolution operations employ a kernel size of 3 × 3 or larger. However, a 3 × 3 or larger convolution performs a weighted summation in the local neighborhood of the input image. Therefore, the pixel value in the output image is inevitably affected by the local neighborhood of the input image. Unlike the 3 × 3 convolutions, the 1 × 1 convolution only maps a single pixel and has nothing to do with the local neighborhood values. That is, it will not be affected by the texture and can keep the source information of inputs. Following this, a fully 1 × 1 convolutional neural network named StainNet is used to extract the mapping relationship from StainGAN. Except for the last convolutional layer, ReLU is used as a convolutional layer to enhance the non-linear mapping ability. Considering the balance of performance and computational efficiency, we used three convolutional layers with 32 channels by default. Therefore, our network only contains about 1,000 parameters, whereas the generator in StainGAN contains millions of parameters.

The training process mainly consists of three steps. Firstly, we trained StainGAN using an unpaired source and target images. Then, the generator of StainGAN was used to normalize the source images. At last, the normalized images were taken as the Ground Truths to train StainNet with L1 Loss and SGD optimizer. The mapping relationship of StainGAN is based on the image content, that is, the mapping relationship will change accordingly with the different image contents. By learning the normalized images by StainGAN, StainNet can transfer the mapping relationship of StainGAN based on image content into a mapping relationship based on pixel values.

## Experiments and Results

In this section, StainNet is compared with the state-of-the-art methods of Reinhard (9), Macenko (11), Vahadane (12), and StainGAN on the cytopathology and histopathology dataset. We report: (1) Quantitative comparison of different methods in the visual appearance, (2) Application results on the cytopathology and histopathology classification task, (3) Quantitative comparison between the whole slide images normalization results and the whole slide images metastasis detection results.

### Evaluation Metrics

In order to evaluate the performance of different methods, we measured the similarity between the normalized image and the target image, and the consistency between the normalized image and the source image.

Two similarity metrics—Structural Similarity index (SSIM) (21) and Peak Signal-to-Noise Ratio (PSNR)—are used to evaluate the performance. The SSIM and PSNR of the target image (SSIM Target and PSNR Target) are used to evaluate the similarity between the normalized image and the target image. The extent of source information preservation is weighed by the SSIM of the source image (SSIM Source), which also was used to measure the similarity between the normalized image and the source image. SSIM Target and PSNR Target are calculated using the original RGB values. SSIM Source is used to measure the preservation of the source image texture information, similar to (22), we used grayscale images to calculate SSIM Source. And the statistic results of SSIM Target, PSNR Target, and SSIM Source on the testing set in the aligned cytopathology dataset and the aligned histopathology dataset are shown in Tables 1, 2, which contain 966 and 7,936 patch pairs, respectively.

**Table 1:** Evaluation metrics of various stain normalization methods on the cytopathology dataset.

**Methods**	**SSIM target**	**PSNR target**	**SSIM source**	**FPS**
Reinhard	0.739 ± 0.046	19.8 ± 3.3	0.885 ± 0.042	54.8
Macenko	0.731 ± 0.054	22.5 ± 3.1	0.853 ± 0.054	4.0
Vahadane	0.739 ± 0.050	22.6 ± 3.0	0.867 ± 0.050	0.5
StainGAN	0.764 ± 0.030	29.7 ± 1.6	0.905 ± 0.021	19.6
StainNet	0.809 ± 0.027	29.8 ± 1.7	0.945 ± 0.025	881.8

**Table 2:** Evaluation metrics of various stain normalization methods on the histopathology dataset.

**Methods**	**SSIM target**	**PSNR target**	**SSIM source**
Reinhard	0.617 ± 0.106	19.9 ± 2.1	0.964 ± 0.031
Macenko	0.656 ± 0.115	20.7 ± 2.7	0.966 ± 0.049
Vahadane	0.664 ± 0.116	21.1 ± 2.8	0.967 ± 0.046
StainGAN	0.706 ± 0.099	22.7 ± 2.6	0.912 ± 0.025
StainNet	0.691 ± 0.107	22.5 ± 3.3	0.957 ± 0.007

The Area Under the Curve (AUC) of the Receiver Operating Characteristics (ROC) is used to evaluate the classifier performance. The statistic results of AUC on the cytopathology and histopathology datasets are shown in Table 3, as Mean ± standard deviation, which contain 6,535 and 10,000 patches, respectively.

**Table 3:** The AUC for various stain normalization methods on the cytopathology and the histopathology classification dataset.

**AUC**	**The cytopathology classification dataset**	**The histopathology classification dataset**
Original	0.832 ± 0.016	0.685 ± 0.033
Reinhard	0.738 ± 0.014	0.821 ± 0.005
Macenko	0.872 ± 0.006	0.843 ± 0.007
Vahadane	0.832 ± 0.011	0.847 ± 0.005
StainGAN	0.896 ± 0.002	0.905 ± 0.006
StainNet	0.901 ± 0.002	0.895 ± 0.009

### Implementation

For conventional methods, Reinhard (9), Macenko (11), and Vahadane (12), a carefully picked image was used as the reference image. For the StainGAN, the model was trained using Adam optimizer, and training was stopped at the 100th epoch, which was chosen experimentally. For StainNet, the trained StainGAN was used to normalize the source images in both the training dataset and the test dataset. Then, the normalized images were used as the ground truths during training. StainNet was trained with stochastic gradient descent (SGD) optimizer, an initial learning rate of .01, and a batch size of 10. The L1 loss was used to minimize the difference between the output of the network and the normalized image by the trained StainGAN. A cosine annealing scheduler was adopted to decay the learning rate from 0.01 to 0 during 300 epochs. The weights corresponding to the model with the lowest test loss were selected during the training.

On the application task, stain normalization was used as a pre-processing step to increase the performance of the CAD system. A classifier was trained on the cytopathology classification dataset and histopathology classification dataset to prove this. We used a pre-trained SqueezeNet (23) on ImageNet (24) as the classifier and fine-tuned it on the images of the training dataset. The classifier was trained with Adam optimizer, an initial learning rate of 2e-4, and a batch size of 64. Cross-entropy loss was used as our loss function. A cosine annealing scheduler was adopted to decay the learning rate from 2e-3 to 0 in 60 epochs. The training was stopped at the 60th epoch, which was chosen experimentally. The experiment was repeated 20 times in order to enhance reliability.

### Results

#### Stain Transfer Results

**Figure 2:**
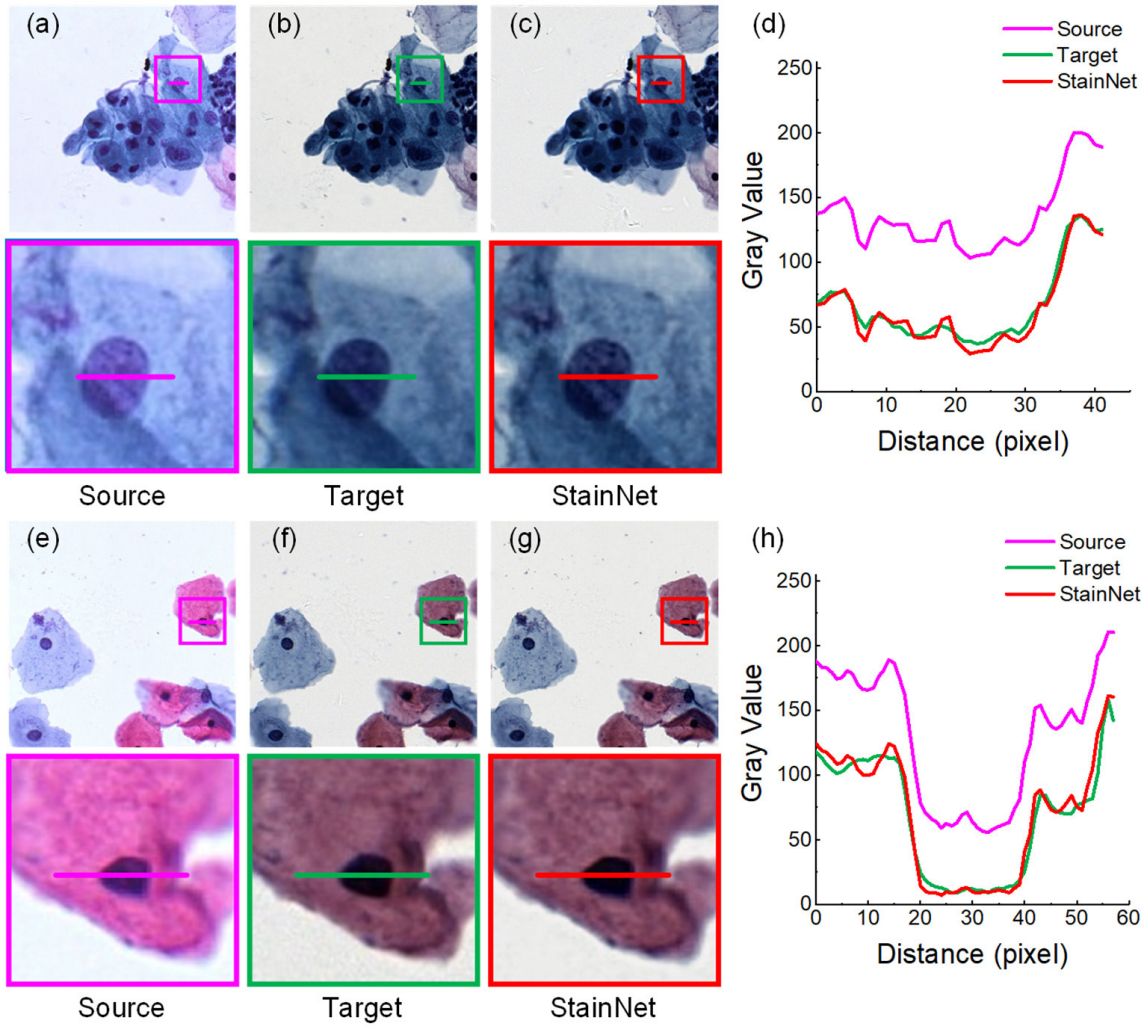
StainNet normalization effects on the cytopathology image. The source images, the target images, and the normalized images by StainNet are shown in **(a,e)**, **(b,f)**, and **(c,g)**, respectively. The image in the box is enlarged below. Gray value profiles of the lines on **(a–c)** are shown in the line chart **(d)**, and the lines in **(e–g)** are shown in the line chart **(h)**.

**Figure 3:**
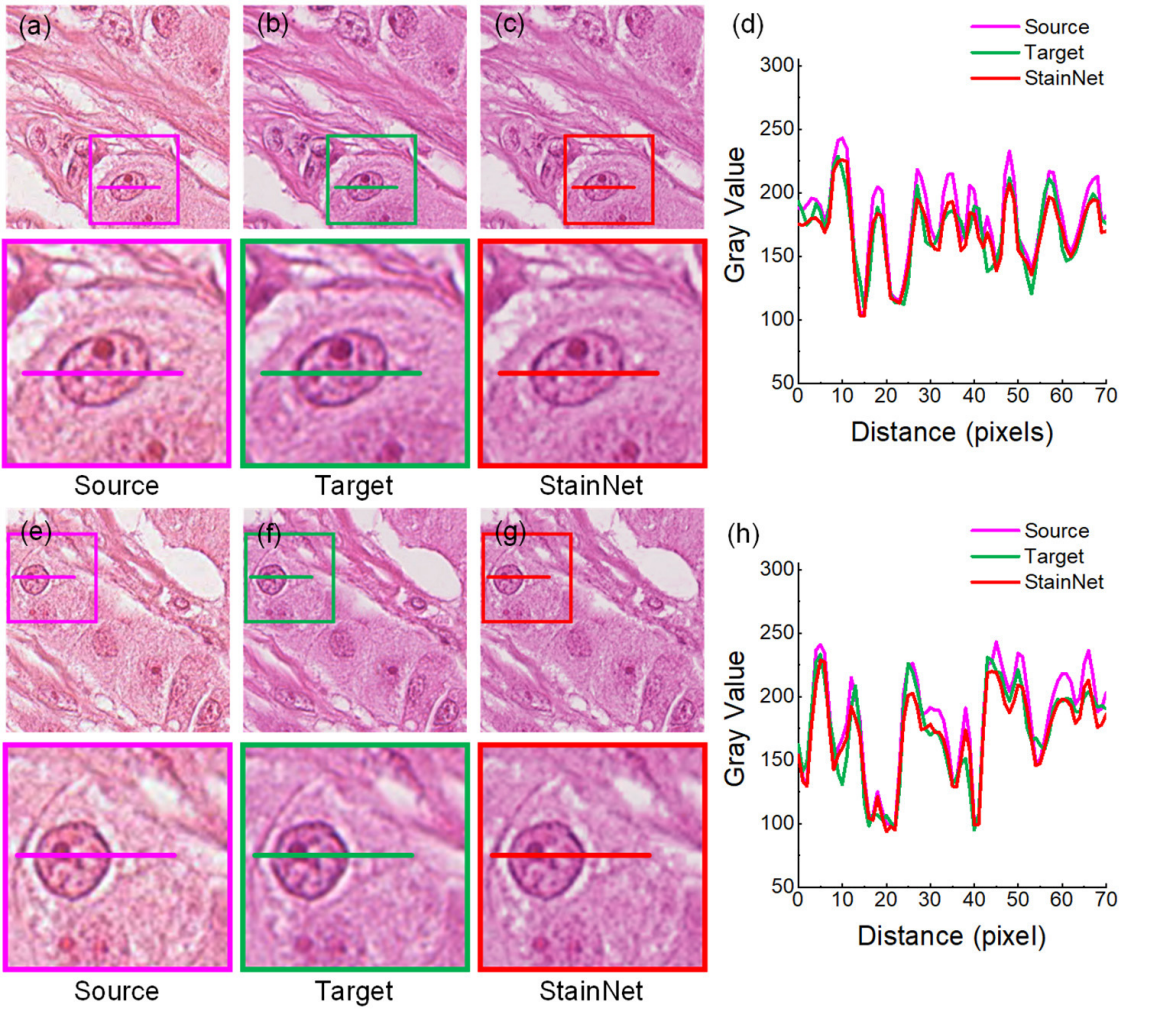
StainNet normalization effects on the histopathology image. The source images, the target images, and the normalized images by StainNet are shown in **(a,e) (b,f)**, and **(c,g)**, respectively. The image in the box is enlarged below. Gray value profiles of the lines on **(a–c)** are shown in the line chart **(d)**, and the lines in **(e–g)** are shown in the line chart **(h)**.

Firstly, we evaluated the effectiveness of our method. The normalized images by StainNet are evaluated with the target images through vision and the gray value profiles around the cell nucleus shown in Figures 2, 3. The results on the aligned cytopathology dataset are shown in Figure 2, the source images are from scanner O, and the target images are from scanner T. From the figure, the normalized images in Figures 2c,g are similar to the target images in Figures 2b,f. The gray value profiles at the nucleus of the source images, target images, and normalized images are shown in Figures 3d,h. The gray value profiles of the normalized images by StainNet and the target images coincide on the whole indicating that, after being normalized by StainNet, the normalized images have similar color distribution with the target images. In terms of local gray value profiles, the changing trend of the normalized images by StainNet is the same as that of the source images, which shows that StainNet can fully retain the information of the source images.

The results on the aligned histopathology dataset are shown in Figure 3. The histopathology dataset was from the publicly available part of the MITOS-ATYPIA ICPR'14 challenge (20). The aligned images are taken from the same slide but using two slide scanners: Aperio Scanscope XT called scanner A and Hamamatsu Nanozoomer 2.0-HT called scanner H. From the figure, we can see, after normalization, the images have a similar vision and the gray value profiles with the target images.

**Figure 4:**
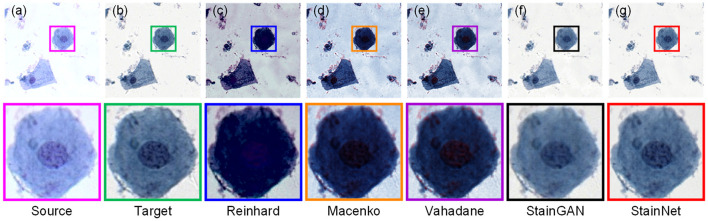
Visual comparison of different normalization methods on the aligned cytopathology dataset. Source image **(a)**, target image **(b)**, and normalized image by Reinhard **(c)**, Macenko **(d)**, Vahadane **(e)**, StainGAN **(f)**, and StainNet **(g)** are listed.

Furthermore, we compare the normalization effect of StainNet with the other four classic methods, Reinhard, Macenko, Vahadane, and StainGAN. Results are shown in Figure 4. From the figure, we can see the Reinhard method performs badly because it is hard to choose an image to represent the entire dataset due to the discreteness of cytopathological images. Macenko and Vahadane, based on stain separation, perform poorly on cytopathological images. Both StainGAN and StainNet perform well.

The quantitative results on the aligned cytopathology dataset are shown in Table 1. From Table 1, parameters PSNR Target of the conventional methods is lower than that of StainGAN and StainNet. StainNet outperforms other methods in all indicators. Among them, SSIM Target and SSIM Source are 0.809 and 0.945 higher than 0.764 and 0.905 of StainGAN, which shows that StainNet is not only more similar to the target image but also better to retain the source image information.

**Figure 5:**
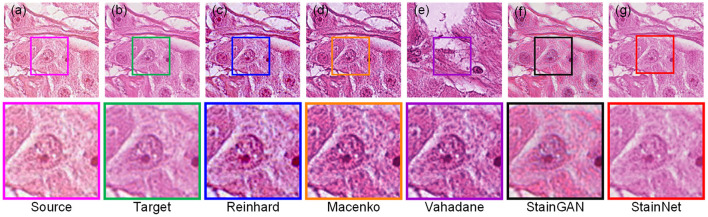
Visual comparison of different normalization methods on the aligned histopathology dataset. Source image **(a)**, target image **(b)**, and normalized image by Reinhard **(c)**, Macenko **(d)**, Vahadane **(e)**, StainGAN **(f)**, and StainNet **(g)** are listed.

The visual comparison of the aligned histopathology dataset is shown in Figure 5. From it, the normalized images by the conventional methods are still visually different from the target image due to the dependence on the reference image and the difficulty of image selection of the conventional methods. The normalized images by StainGAN and StainNet are consistent with the style of the target image. In addition, the normalized image by StainNet not only has a similar color to the target image but also retains more source information.

The quantitative comparison of the aligned histopathology dataset is shown in Table 2. The test data and training data are completely separated at the slide level and divided in the same way as in the MITOS-ATYPIA ICPR'14 challenge (20), so there is no deviation caused by personal factors. Due to the rigid and non-rigid registration, the source image and the target image can be precisely matched. The dataset division and image registration make our results more reliable. StainGAN and StainNet are higher than conventional methods in the similarity of SSIM Target and PSNR Target with the target images. The SSIM Target and PSNR Target of StainNet are 0.691 and 22.5, respectively, which are slightly lower than 0.706 and 22.7 of StainGAN, 0.957 of StainNet is higher than 0.912 of StainGAN in the SSIM Source. Therefore, StainNet can obtain normalized results comparable to StainGAN but retain the source image information better, which is important in real CAD systems.

Next, we compared the normalization effects StainNet and StainGAN on image classification. SqueezeNet (22) pre-trained on ImageNet (23) was chosen as the classifier because of its small size and relatively high accuracy. On the cytopathology classification dataset, we used 13,178 image patches from scanner T to train the classifier and use 6,535 image patches from scanner O to evaluate the classifier. On the histopathology classification dataset, the classifier was trained with 40,000 image patches from Radboud University Medical Center, and the classifier was evaluated with 10,000 image patches from University Medical Center Utrecht. Table 3 shows the performance of the classifier with normalization and not with normalization. For the original images in the test set, there is only an AUC of 0.832 on the cytopathology classification dataset, and only 0.685 on the histopathology classification dataset. It shows that the classifier has a strong color bias and cannot be directly applied to the test data with different color styles from the training data. The AUC was increased to 0.896 and 0.905 by using StainGAN and 0.901 and 0.895 by using StainNet on the cytopathology classification dataset and histopathology classification dataset. The conventional stain normalization methods hardly achieve a better AUC, especially in the histopathology classification dataset, and the performance of the conventional method is lower than StainGAN and StainNet. The above results show that both StainGAN and StainNet can effectively improve the accuracy of the classifier, and the performance of the StainNet method and the StainGAN method is comparable.

#### Whole Slide Images Results

For a whole slide image (WSI), there are two main challenges in stain normalization: One is that WSIs are very large: a typical WSI may contain 100,000 × 100,000 pixels. So, computational efficiency is very important. The other is that WSIs may contain many naturally occurring and human-induced artifacts, e.g., air bubbles, dust, and out-of-focus. So, the methods must be robust to these phenomena when they are applied in a real-world system. Since StainNet has a very concise structure and only maps based on color values, it is less affected by the distribution of the training data and has better robustness.

In this experiment, we randomly selected 20 cytopathology WSIs from the same data source in section The Aligned Cytopathology Dataset for Evaluating the Similarity and 20 histopathology WSIs from the Camelyon16 dataset. StainGAN and StainNet are used to normalize these WSIs to the target style. Results show the computational efficiency of StainNet is more than 40 times that of StainGAN and can normalize a 100,000 × 100,000 whole slide image in 40 s, which is very important for real-time application.

**Figure 6:**
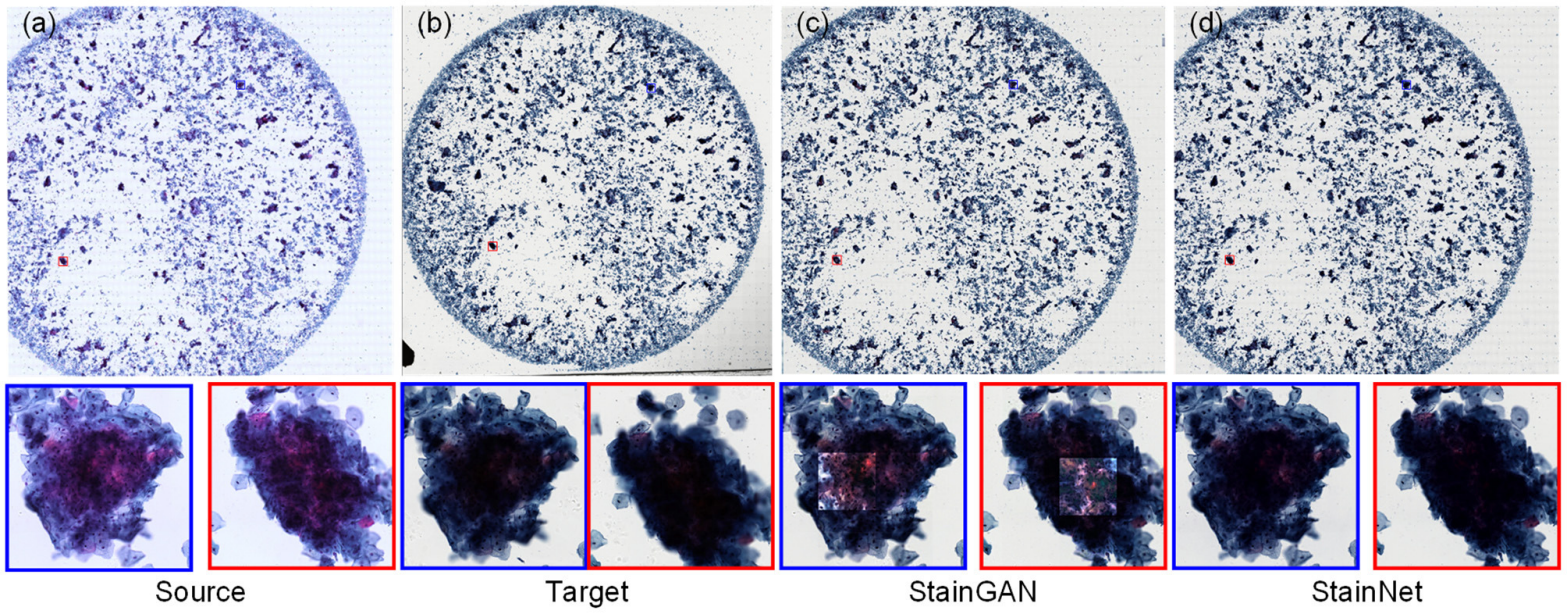
The whole slide image normalization result on the cytopathology dataset. The source slide **(a)**, the target slide **(b)**, the normalized slide by StainGAN **(c)**, and the normalized slide by StainNet **(d)** are listed.

For the cytopathology WSIs, StainGAN and StainNet trained on the aligned cytopathology dataset were used to perform normalization, as shown in Figure 6. From Figure 6, the normalized WSI by StainGAN has artifacts in the center of crowded cell clusters. Our proposed StainNet achieves better results maybe because of its robustness and less reliance on the distribution of the training set.

**Figure 7:**
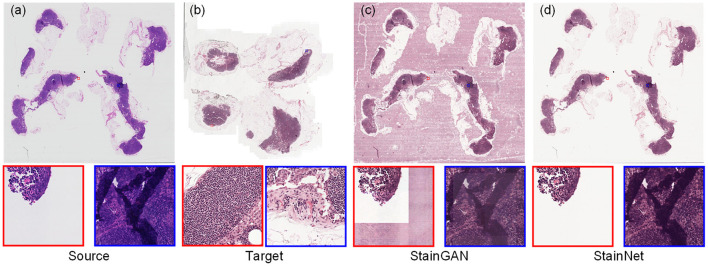
The whole slide image normalization result on the Camelyon16 dataset. The source slide **(a)**, the target slide **(b)**, the normalized slide by StainGAN **(c)**, and the normalized slide by StainNet **(d)** are listed.

For the histopathology WSIs, StainGAN and StainNet trained on the histopathology classification dataset were used to perform normalization, as shown in Figure 7. StainGAN has artifacts in the blank background area and the out-of-focus area. Similar to the cytopathology WSIs, StainNet achieves a better performance.

SSIM Source was used to quantitatively evaluate the normalized performance by StainGAN and StainNet in this experiment. In Table 4, StainNet has a higher mean value and a lower standard deviation, which shows that StainNet not only can obtain better image quality but also has consistent and robust performance on the WSIs. The standard deviation of StainGAN is increased, which shows that the performance of StainGAN is not stable enough on the WSIs.

**Table 4:** The SSIM source of the normalized whole slide image by StainGAN and StainNet.

	**StainGAN**	**StainNet**
The cytopathology WSIs	0.905 ± 0.093	0.954 ± 0.050
The histopathology WSIs	0.762 ± 0.182	0.980 ± 0.013

**Figure 8:**
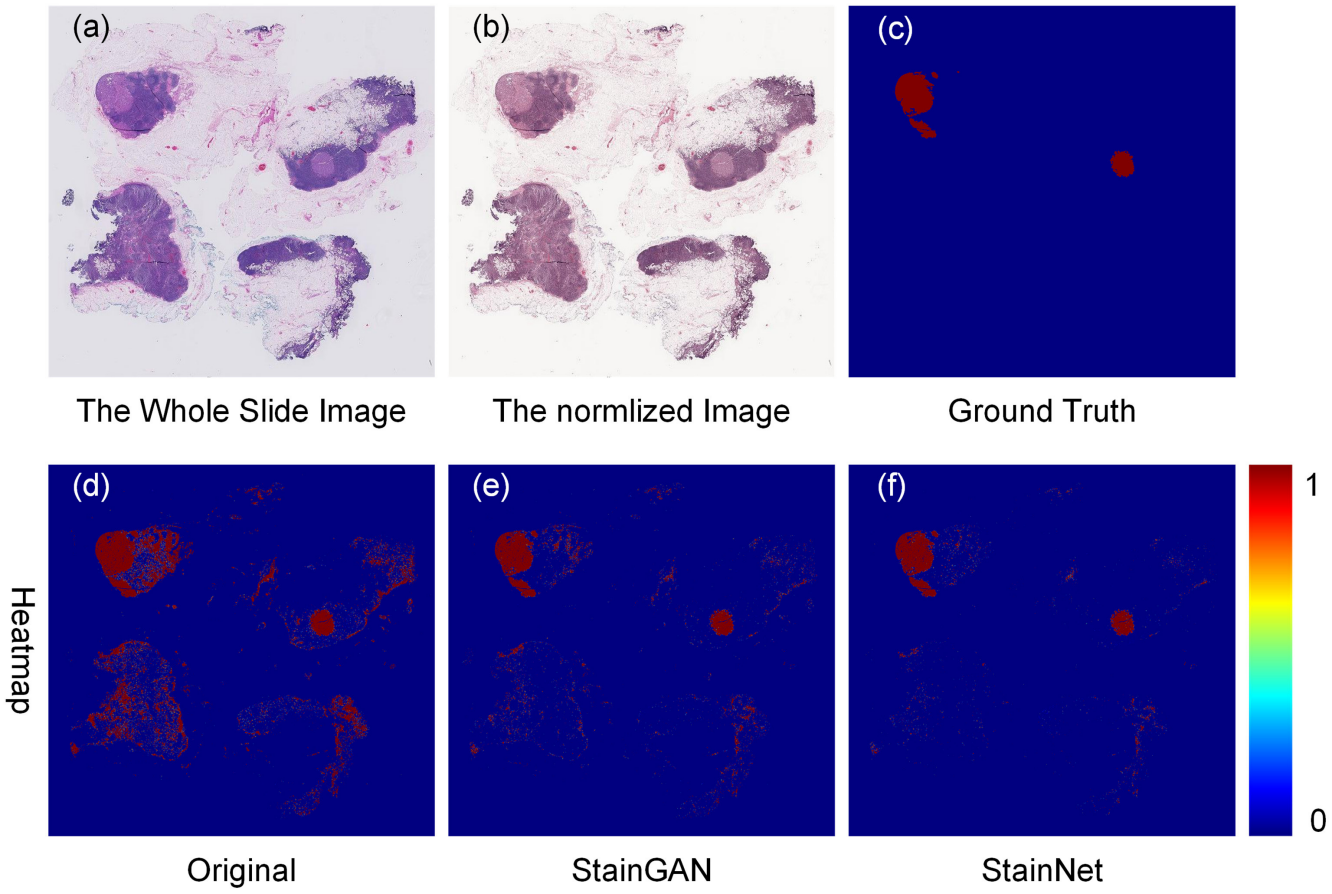
Effects of color normalization on metastasis cancer tissue detection. The original whole slide image **(a)**, the normalized image by StainNet **(b)**, the ground truth **(c)** of the metastasis cancer, and the detection heat maps from the original image **(d)**, from the image by normalized StainGAN **(e)**, and from the image by normalized StainNet **(f)**.

Furthermore, we tested the effects of the StainNet normalization on clinical diagnostics. Here, we chose the metastasis cancer slides and demonstrated the detection results on the WSI level. Twenty metastases WSIs from the University Medical Center Utrecht in Camelyon16 testing part were used, and the whole slide image was divided into several 256 × 256 image blocks with a 64 × 64 stride by a sliding window way. SqueezeNet was trained on the histopathology classification dataset, and then to detect the original WSIs and the normalized WSIs by StainGAN and StainNet, shown in Figure 8. From the picture, we can see, compared with the grand truth, there are a large number of normal areas that are misidentified as metastasis areas. After being normalized with StainGAN and StainNet, the misidentification area is reduced. The statistic results are shown in Table 5; the parameters of recall, precision, and accuracy are used to quantitatively evaluate the metastasis detection results on the WSIs. It can be seen that the precision of the original WSIs is only 0.368, and StainGAN and StainNet can improve the precision of recognition, which are 0.708 and 0.781, respectively. For the accuracy of recognition of all image blocks on the whole slide image, the accuracy of the original image without normalization is 0.940, and the accuracy of StainGAN and StainNet is 0.977 and 0.979, respectively. The preliminary results show that our method is better than StainGAN in accuracy and precision in the application of WSI metastasis detection.

**Table 5:** The recall, precision, and accuracy of the metastasis detection at the WSI level.

	**Recall**	**Precision**	**Accuracy**
The original WSIs	0.781	0.368	0.940
The normalized WSIs by StainGAN	0.686	0.708	0.977
The normalized WSIs by StainNet	0.629	0.781	0.979

**Figure 9:**
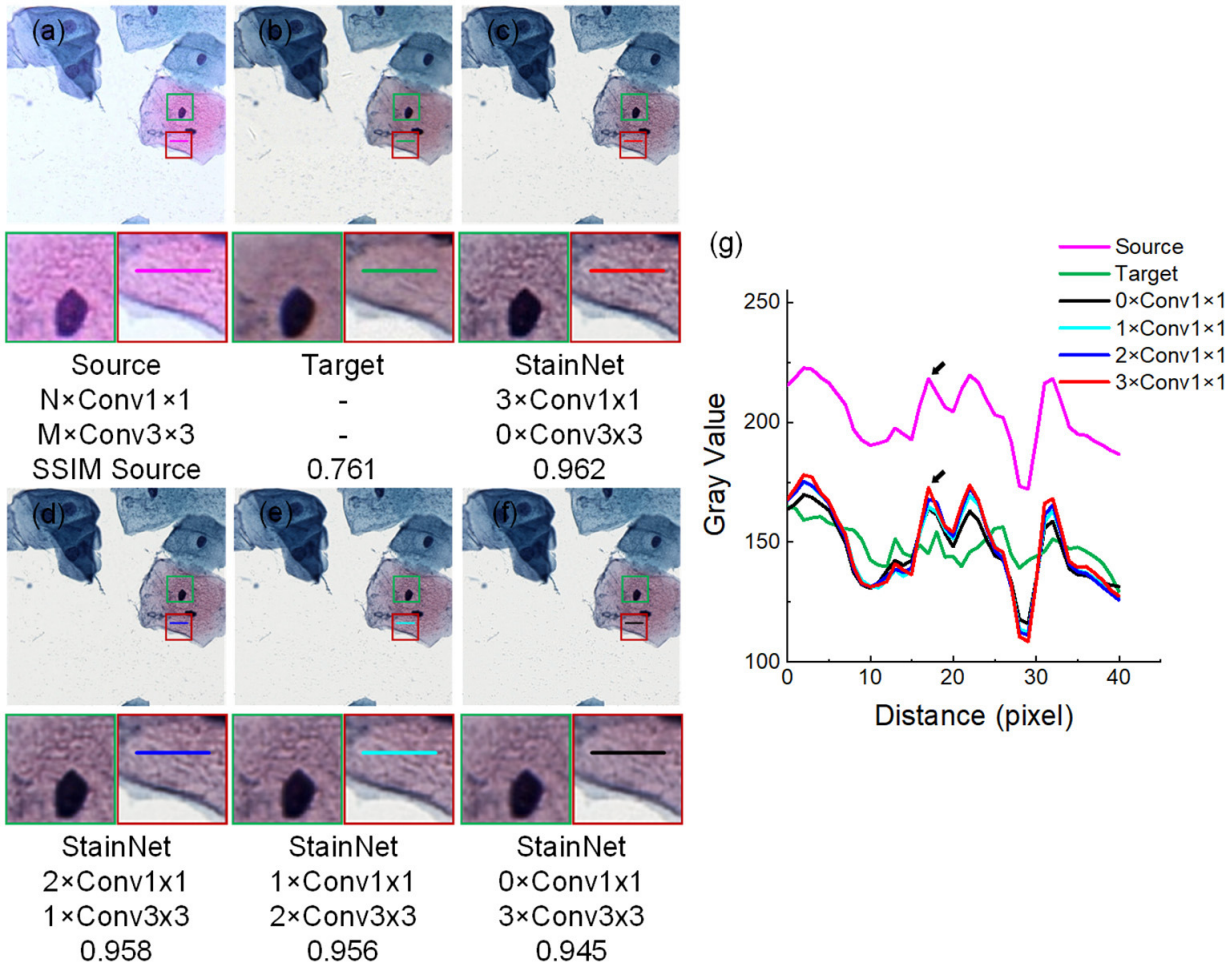
Effects of 1 × 1 and 3 × 3 convolutions. NxConv1x1 and MxConv3x3 refer to the number of 1 × 1 convolution and 3 × 3 convolutions. StainNet contains only three convolution layers, so the total number of 1 × 1 convolution and 3 × 3 convolutions is three, that is, M + N = 3. The image in the dashed box is enlarged below. Gray value profiles of the straight lines in **(a–f)** are shown in the line chart **(g)**.

The effectiveness of 1 × 1 convolution is verified by replacing the three 1 × 1 convolutions in StainNet with 3 × 3 convolutions in turn. The source image, target image, and normalized image by different structures of StainNet are shown in Figures 9a–f, and the gray value profiles of the straight lines in Figures 9a–f are shown in Figure 9g. It is clear that, with the increase of 3 × 3 convolutions, the normalized image becomes more blurred, and the ability to preserve the source information is getting worse. The best image quality can be obtained fully using 1 × 1 convolution in Figure 9c. In particular, at the place pointed by the black arrow in Figure 9g, only a fully 1 × 1 convolutional network can best preserve the grayscale changes of the source image.

The different evaluation metrics, SSIM Target, PSNR Target, and SSIM Source for different structures of StainNet are reported in Table 6. Although the 3 × 3 convolutions may help improve the similarity with the target images, they affect the ability to preserve the source information. Not changing the information of the source image is a basic requirement for stain normalization, so a fully 1 × 1 convolutional network is chosen.

**Table 6:** Evaluation metrics of different StainNet structures.

**Number of Conv 1 x 1**	**Number of Conv 3 x 3**	**SSIM target**	**PSNR target**	**SSIM source**
3	0	0.808	29.8	0.960
2	1	0.814	30.0	0.958
1	2	0.814	30.0	0.956
0	3	0.804	29.8	0.950

## Discussion and Conclusion

In this paper, we achieved stain normalization by using a fully 1 × 1 convolutional network in a pixel-to-pixel manner, which not only avoids the low computational efficiency and possible artifacts of deep learning-based methods but also preserves well the information of the source image. Compared with conventional methods, StainNet learns the mapping relationship from the whole dataset instead of relying on one single reference image, so it can obtain the normalized image with high similarity. Furthermore, StainNet has been validated on four datasets, including two public datasets, and the results show that StainNet has better performance, especially in computational efficiency and robustness.

Compared with the traditional methods, StainNet avoids the difficulty of choosing reference images. For the cytopathy image, the proportion of blank backgrounds is various, so the standard deviation and mean of the different images also are different, and we cannot find an image to represent the entire dataset. This is the reason that the Reinhard method does not perform well in Figure 4c. For Macenko and Vahadane, the color normalization method is based on stain separation, it is difficult to perform stain separation correctly due to the use of multiple stains for cytopathological images instead of only eosin and hematoxylin in histopathology.

Compared with StainGAN, StainNet achieves comparable normalization performance. At the same time, StainNet is more than 40 times that of StainGAN in the computational efficiency and can normalize a giga-pixel WSI in 40 s. And, more importantly, StainNet retains the source information better and would not produce some artifacts. StainNet retains the advantages of better color normalization of StainGAN, while a fully 1 × 1 convolutional network overcomes the determination of slow speed and instability.

In short, StainNet, a fast and robust stain normalization network, has the potential to perform normalization in real-time in a real-world CAD system.

## Data Availability Statement

The datasets presented in this study can be found in online repositories. The names of the repository/repositories and accession number(s) can be found at: https://github.com/khtao/StainNet.

## Author Contributions

HK contributed to the conception, implemented the experiments, and wrote the first draft of the manuscript. JH provided a cytopathology dataset and annotated the cytopathology images. HK, DL, WF, JH, SZ, TQ, and XL designed the study and contributed to the result analysis and manuscript revision. All authors approved the manuscript.

## Funding

This study was supported by National Natural Science Foundation of China (NSFC) (61721092), Director Fund of Wuhan National Laboratory for Optoelectronics, and Research Fund of Huazhong University of Science and Technology.

## Conflict of Interest

The authors declare that the research was conducted in the absence of any commercial or financial relationships that could be construed as a potential conflict of interest.

## Publisher's Note

All claims expressed in this article are solely those of the authors and do not necessarily represent those of their affiliated organizations, or those of the publisher, the editors and the reviewers. Any product that may be evaluated in this article, or claim that may be made by its manufacturer, is not guaranteed or endorsed by the publisher.
